# Effects of Albumin–Chlorogenic Acid Nanoparticles on Apoptosis and PI3K/Akt/mTOR Pathway Inhibitory Activity in MDA-MB-435s Cells

**DOI:** 10.3390/nano13091438

**Published:** 2023-04-22

**Authors:** Badr Alzahrani, Abozer Y. Elderdery, Abdullah Alsrhani, Nasser A. N. Alzerwi, Maryam Musleh Althobiti, Musaed Rayzah, Bandar Idrees, Ahmed M. E. Elkhalifa, Suresh K. Subbiah, Pooi Ling Mok

**Affiliations:** 1 Department of Clinical Laboratory Sciences, College of Applied Medical Sciences, Jouf University, Sakaka 72388, Saudi Arabia; 2 Department of Surgery, College of Medicine, Majmaah University, P.O. Box 66, Al-Majmaah 11952, Saudi Arabia; 3 Department of Clinical Laboratory Science, College of Applied Medical Science, Shaqra University, Shaqra 11961, Saudi Arabia; 4 Department of Surgery, Prince Sultan Military Medical City in Riyadh, Makkah Al Mukarramah Rd, As Sulimaniyah 12233, Saudi Arabia; 5 Department of Public Health, College of Health Sciences, Saudi Electronic University, Riyadh 11673, Saudi Arabia; 6 Department of Haematology, Faculty of Medical Laboratory Sciences, University of El Imam El Mahdi, Kosti 1158, Sudan; 7 Centre for Materials Engineering and Regenerative Medicine, Bharath Institute of Higher Education and Research, Chennai 600073, India; 8 Department of Biomedical Science, Faculty of Medicine & Health Sciences, Universiti Putra Malaysia, Serdang 43400, Malaysia

**Keywords:** MDA-MB435s cells, albumin, chlorogenic acid, biogenic nanoparticles, apoptosis, anti-cancer

## Abstract

In this study, we synthesized, characterized, and explored the anti-microbial and anti-cancer effects of albumin–chlorogenic acid nanoparticles (NPs). Characterization studies with a UV-vis spectrophotometer, FTIR, PL spectrum, TEM, FESEM, XRD, and DLA analysis showed patterns confirming the physio–chemical nature of biogenic nanocomposites. Further, anti-microbial studies using bacterial strains *Staphylococcus aureus*, *Streptococcus pneumonia*, *Bacillus subtilis*, *Escherichia coli*, *Pseudomonas aeruginosa*, *Vibrio cholera*, and fungal strain *Candida albicans* showed significant (*p* < 0.05) anti-bacterial and anti-fungal activities. Next, we used MDA-MB-435s, a human cell line, to evaluate the anti-cancer effects of albumin–chlorogenic acid NPs. Cytotoxic studies revealed its IC50 concentration at 24 μg/mL after a 24 h treatment of MDA-MB-435s cells. We chose this IC50 dose to analyze albumin–chlorogenic acid NPs anti-cancer effects in vitro. MDA-MB-435s cells exposed to our NPs were studied via AO/EtBr staining, cell cycle analyses via PI staining, the status of whole genomic damage via comet assay, levels of apoptotic cells via annexin V/PI staining, ROS generation via DCFH-DA staining, an assay of antioxidant enzymes catalase, superoxide dismutase, and antioxidant GSH, via ELISA analyses of apoptotic markers caspase-3, 8, 9, Bax, Bcl-2, CytC, and p53, PI3/AKT/mTOR pathway. Our results collectively showed albumin–chlorogenic acid NPs induced apoptosis via p53-dependent and PI3/AKT/mTOR inhibition in MDA-MB-435s cells. Our results denote albumin–chlorogenic acid NPs can be used as an effective candidate for anti-microbial and anti-cancer applications; however, further in vivo confirmatory studies are warranted.

## 1. Introduction

In recent decades, immense developments in the field of nanoscience have contributed to its applications in various fields such as medicine, engineering, environmental studies, etc., [1]. In particular, the role of nanoscience in the development of nanomedicine drug development has imparted new hope to many patients suffering from incurable diseases like cancers, and neurodegenerative diseases such as Alzheimer’s, Parkinson’s, etc. [2,3]. In this scenario, the development of green nanotechnology, that is, development/synthesis of green nanoparticles (NPs) is currently a hot topic due to its enormous applications in the biomedical field (theragnostic drug delivery, antimicrobial, tissue engineering, bioimaging, nano vaccines, etc.), food, and other fields such as agriculture [4,5]. Nowadays, researchers around the world use a green synthesis of NPs for their advantages like synthesis using natural reduction, capping, stabilizing, and devoicing use of harmful chemicals (toxic to humans and the environment), and minimizing the requirement for high energy for synthetic NPs [6,7]. In the green synthesis approach, various plant parts like leaves, roots, flowers, and fruit and microorganisms like fungi, bacteria, algae, yeast, etc., are used for the NPs synthesis. Contrary to the chemical reduction in NPs synthesis, green synthesis of NPs use reducing agents like amino acids, citric acid, aldehyde, flavonoids, secondary metabolites, etc., [7].

Chlorogenic acid (CGA) nanoparticles refer to tiny particles composed of chlorogenic acid molecules that have been assembled together. Nanoparticles are typically sized between 1 and 100 nanometers in diameter. This is extremely small, making them highly useful in various applications such as drug delivery, cosmetics, and the food industry [8]. Chlorogenic acid is a type of polyphenol commonly found in many plant species, including coffee, apples, pears, and blueberries. It has been found to possess an array of pharmacological benefits, including antioxidant and anti-inflammatory properties, and has been investigated for its potential to treat various diseases such as diabetes and cardiovascular disease [9]. The use of CGA nanoparticles allows for increased bioavailability and targeted delivery of chlorogenic acid to specific tissues or organs in the body. Additionally, the small size of the nanoparticles permits them to be effortlessly absorbed into the body, which can enhance the therapeutic effects of chlorogenic acid [10]. CGA nanoparticles can be synthesized using various methods, including chemical and physical methods. They can be further modified with different coatings or functional groups to improve their stability and properties. They have potential applications in fields such as nanomedicine, functional foods, and nutraceuticals [11].

Previous studies reported that 1,2,4-trioxanes are used in antitumor works to decrease osteopontin (OPN) levels in MDA-MB-435s cells. The synthetic 1,2,4-trioxanes have been shown, and 1,2,4-trioxane dimers display notable reduction in OPN and may act as antimetastatic agents. [12]. A previous study found that *Psidium guajava* pulp extract had the highest in vitro antioxidative and antiproliferative activity in breast carcinoma cells, as well as the highest levels of lycopene and total phenolics. A number of guava cultivars’ extracts decreased the MDA-MB-435s cell growth and increased apoptosis in MCF-7 cells [13,14]. There was a significant difference between the cytotoxicity of dehydroberberine derivatives against different types of cancer cells (e.g., MDA-MB-435s, MDA-MB-468, U87MG, HeLa, MDA-MB-231, MCF-7, and HCT116 cells) compared with non-malignant tissues (e.g., HUVEC, HEK293) [15]. According to a previous study, ethanolic extracts of *Tinospora cordifolia* inhibited ABC transporters in both HeLa and MDA-MB-435s cells. However, compared to the ethanolic extract, extracts of petroleum ether and dichloromethane fractions showed a more than 90% reduction in the cell population of HeLa and MDA-MB-435s cells [16]. In previous studies, it has been shown that apoptosis induction potentials were found for ethanolic and aqueous *R emodi* rhizome extracts in MDA-MB-435s, Hep3B, and PC-3 cells examined by ELISA to identify DNA fragmentation [17]. In the present study, the cytotoxic potential of *Annona muricata* L. leaves was evaluated by using WRL-68 cells, MDA-MB-435s cells, and HaCaT cells [18].

Our study hypothesized that a novel albumin–CGA nanoparticle would be synthesized and characterized using XRD, FTIR, PL spectroscopy, and TEM. Our results also revealed that albumin–CGA nanoparticles possess significant anti-microbial and anti-cancer properties during in vitro experiments with MDA-MB-435s cells. The in vivo biological functions of this compound are being evaluated in further studies.

## 2. Materials and Methods

### 2.1. Chemicals and Their Sources

Albumin, chlorogenic acid (CGA), alcohol, acridine orange, ethidium bromide (EtBr), bovine serum albumin (BSA), sterile coverslips, tissue culture plates, MTT, DMSO, propidium iodide, Annexin V kit, Comet assay kit, ELISA kits, antibiotics for cell culture (penicillin/streptomycin), sterile PBS, Ca2+, Mg2+ free PBS, RIPA buffer, trypsin/versene, BCA Kit, DCFH-DA kit, and other analytical grade chemicals were acquired from Merck, Darmstadt, Germany and Abcam chemical company, Waltham, USA. Bacterial cultures and MDA-MB-435s cell lines were purchased from NCCS, Pune, India.

### 2.2. Synthesis of Albumin–Chlorogenic Acid Green NPs

Albumin–CGA green NPs were synthesized using standard protocols as reported earlier with slight modifications [17,18]. In brief, 50 mg of chlorogenic acid was added with 500 mg of albumin in deionized water (20 mL) for 6 h at 37 °C, then mixed with 100 µL glutaraldehyde (0.5%). Afterward, the unreactive portions were discarded by dialyzing in water for 1 day, ensuing in albumin–chlorogenic acid NPs.

### 2.3. Characterization of Albumin–Chlorogenic Acid Green NPs

The albumin–chlorogenic acid NPs obtained were subjected to physio–chemical analyses via X-ray diffractometer (XRD) (model: X’PERT PRO PANalytical). The diffraction patterns were recorded in the 2θ range of between 25° and 80° for albumin–chlorogenic acid NPs, with a monochromatic CuKα diffraction beam of 1.5406 Å wavelength. A FE-SEM (Carl Zeiss Ultra-55 FE-SEM) with EDX spectrometry (model: Inca) was employed to investigate albumin–chlorogenic acid NPs. The morphologies of the albumin–chlorogenic acid NPs were analyzed by TEM (Tecnai-F20) machine, which was operated at 200 kV accelerating voltage. The spectrum of the FTIR (Perkin-Elmer) was noted in the wave number of 400–4000 cm^−1^. The Lambda-35 spectrometer was employed to detect the absorption spectrum of albumin–chlorogenic acid NPs at 200 and 1100 nm. Photoluminescence (PL) spectrum was acquired using a spectrometer [19].

### 2.4. Analysis of Anti-Bacterial, Anti-Fungal Effects of Albumin–Chlorogenic Acid Green NPs

For the antibacterial studies, we used the albumin–CGA NPs at concentrations ranging from 1, 1.5, 2 mg/mL and standard antibiotic amoxicillin/amphotericin-B. Antibacterial studies were performed with the agar plates streaking methods using bacterial cultures *Staphylococcus aureus*, *Streptococcus pneumonia*, *Bacillus subtilis*, *Escherichia coli*, *Pseudomonas aeruginosa*, *Vibrio cholera*, and fungal strain *Candida albicans* based on the previously reported methods with slight modifications. Via the well-diffusion method, antibacterial activity targets against microorganisms include gram-positive and gram-negative pathogens. In this study, the fabricated Petri plate was loaded with 25 mL of agar medium, and then pathogens were separately inoculated. The albumin–CGA NPs at 1, 1.5, and 2 mg/mL, dissolved in a 5% DMSO. In addition, the plates were sustained for 24hr at 37 °C, and the zones of inhibition was noted. The standard antibiotic amoxicillin (10 μg) for bacterial and amphotericin B (10 μg) fungal was utilized as the positive control. Experiments were carried out in triplicate [20].

### 2.5. Invitro Anti-Cancer Studies

#### 2.5.1. Cell Viability Assay

MDA-MB-435s cells were cultivated at 5% CO_2_ and 37 °C, in an DMEM medium enriched with 1% antimycotic cocktail and 10% FBS in a humidified sterile incubator. To determine the invitro cytotoxicity of albumin–CGS NPs, a cell viability test was performed using an MDA-MB-435s cell line (1 × 10^4^ cells/well in 96-well plates). The following concentrations were used: 3, 6, 12, 24, 48, 96, and 192 μg/mL. A cell viability assay was performed using standard protocols as reported earlier with slight modifications [21].

#### 2.5.2. Acridine Orange/Ethidium Bromide Staining

The MDA-MB 435s cell line (2 × 10^4^ cells/mL,) was seeded and grown on sterile 6-well plate and then cells were treated with albumin–CGA NPs (ACNPs; IC50 concentration) and paclitaxel (0.5 μM) concentration for 24 h. After that, cells were visualized using a fluorescence microscope following the standard procedure as reported earlier [22].

#### 2.5.3. Cell Cycle Analysis

About 2 × 10^6^ cells were used for cell cycle analysis using FACS. Briefly, the cells were grown on a 10 cm dish for 12 h. Later, the semi-confluent cells were exposed to albumin–CGA NPs (ACNPs; IC50 concentration) and paclitaxel (0.5 μM) for 24 h. Following the treatment schedule, cells were gathered and gently rinsed to make it a single cell suspension and then stained using 50 μg/mL PI for 15–30 min following the standard protocol as reported earlier, and FACS analysis was performed [22].

#### 2.5.4. Comet Assay Analysis

The MDA-MB-435s cells were grown on 25 cm^2^ dishes, and the cells were treated with albumin–CGA NPs (ACNPs; IC50 concentration) and paclitaxel (0.5 μM) concentration for 24 h. Following treatment, the cells were gathered using mild trypsinization, and the cells were gently mixed to get single cell suspensions, and they were counted to obtain a final concentration of 100 cells/0.5mL in 1% normal melting point agarose heated at 37 °C. Then cells along with agar were spread onto clean sterile frosted glass slides and subjected to electrophoresis separation following standard protocols as per the manufacturer’s recommendation [23].

#### 2.5.5. Annexin-V/FITC/PI Flow Cytometry

The MDA-MB-435s (1 × 10^5^ cells/well) cells were grown in a 24-well plate overnight at 5% CO_2_ and 37 °C. After the incubation, cells were treated in a medium consisting albumin–CGA NPs (ACNPs; IC50 concentration) and paclitaxel (0.5 μM) for 48 h. Then cells were gathered and gently suspended to get single cell suspensions and stained using Annexin V/PI for assessment of cell death analysis. FACS analysis was employed following standard procedures as reported earlier with slight modifications [24].

#### 2.5.6. Intracellular ROS Analysis by DCFH-DA

The MDA-MB-435s cells (5 × 10^4^ cells/well) was cultivated in 24-well plates overnight and then cells were treated in a medium containing albumin–CGA NPs (ACNPs; IC50 concentration) and paclitaxel (0.5 μM) concentration for 24 h. After incubation, cells were probed using 2′, 7′-dichlorofluorescin diacetate (DCFH-DA, Molecular Probes) for 15–30 min (10 μM) for 20 min at 37 °C. Then, cells were rinsed and visualized using a fluorescence microscope (Nikon, fluorescence microscope) [25].

#### 2.5.7. Assay of Antioxidant Systems in MDA-MB-435s Cells

MDA-MB-435s cells (2 × 10^6^ cells/well) were grown in 100 mm sterile dishes over-night, and the cells were treated in a medium containing albumin–CGA NPs (ACNPs; IC50 concentration) and paclitaxel (0.5 μM) concentration for 24 h. Following treatment, cells were trepanned and harvested, and used for an assay of total protein [26], cellular antioxidants, and antioxidant enzymes. Assays such as catalase (CAT) [27], superoxide dismutase (SOD) [28], intracellular glutathione (GSH) [29], and lipid peroxidation marker assay (MDA) [30] were performed following standard protocols, and are reported with slight modifications.

#### 2.5.8. ELISA Analysis of Apoptotic Markers

MDA-MB-435s cells (2 × 10^6^ cells/well) were grown in sterile dishes overnight, and the cells were treated in a medium containing albumin–CGA NPs (ACNPs; IC50 concentration) and paclitaxel (0.5 μM) concentration for 24 h. For evaluating the expressions of apoptosis protein markers, like caspase-3, 8, 9 Bax, Bcl-2, Cyt-C, and p53, we used ELISA kits as per the guidelines given by the manufacturer (Abcam, Waltham, USA). Initially, MDA-MB435s cells were treated with 24 μg/mL for 24 hrs using a medium containing albumin–CGA NPs. Then the cells were lysed and added onto the ELISA plates coated with appropriate antibodies specific for each apoptotic protein, and the measurements were done calorimetrically on a 96-well microplate reader. This method was performed with slight modifications from previous reports [31,32].

#### 2.5.9. RT-PCR Analysis of PI3K/AKT/mTOR Pathway

MDA-MB-435s cells (2 × 10^6^ cells/well) were grown in sterile dishes overnight, and the cells were treated in a medium containing albumin–CGA NPs (ACNPs; IC50 concentration) and paclitaxel (0.5 μM) concentration for 24 h. For evaluating the gene expressions of PI3/AKT/mTOR, we used conventional RT-PCT (reverse transcriptase polymerase chain reaction) GAPDH, forward 5′ CCTCCCGCTTCGCTCTCT 3′, reverse 5′ GCTGGCGACGCAAAAGA 3′; forward: 5′-GTGCTGGAGGACAATGACTACG-3′, AKT, forward: 5′-GTGCTGGAGGACAATGACTACG-3′, reverse: 5′-AGCAGCCCTGAAAGCAAGGA-3′, mTOR forward, AGCATCGGATGCTTAGGAG-TGG, reverse CAGCCAGTCATCTTTGGAGACCPI3K forward: 5′-AACTCTGGGGATGACCTGGA-3′, reverse: 5′-AGGCGGTCACAACACTCCTA-3′; reverse: 5′-AGCAGCCCTGAAAGCAAGGA-3′. Following drug treatment, MDA-MB-435s cells were collected and mRNA was isolated using trizol, complementary DNA (CDNA) was converted, and the specific primers were used to amplify the genes corresponding to PI3/AKT/mTOR signaling pathway in an Eppendorf PCR thermocycler using standard protocols as per manufactures instructions in the PCR kit and with modifications in Tm (melting temperature adjusted in standardization) [33]. GAPDH was employed as an internal control and for normalizing the differences in gene expression levels.

### 2.6. Statistical Analysis

Assays were done in triplicates. Outcomes are assessed using one-way ANOVA and Tukey test using SPSS software, and *p* values less than *p* < 0.05 were considered for statistical significance.

## 3. Results

### 3.1. Characterization of Albumin–Chlorogenic Acid Green NPs

**Figure 1:**
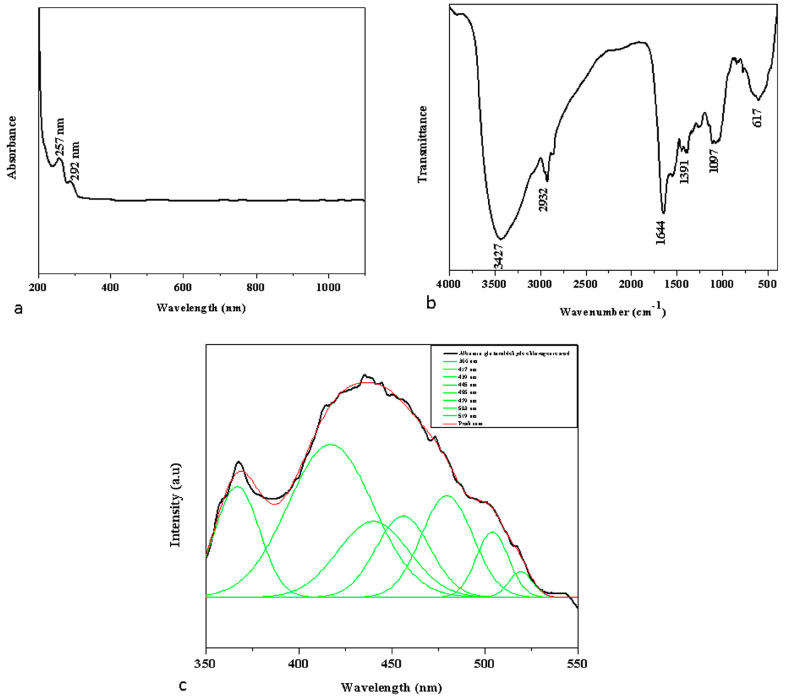
UV-Vis spectrophotometer (**a**), FTIR transmittance vs. wavenumber chart (**b**) and PL spectrum (**c**) study of formulated albumin–chlorogenic acid NPs.

UV-vis spectral analysis (Figure 1a) showed peaks at 257 and 292 nm; FTIR transmittance showed wave patterns at the following stages: 3427 cm^−1^, 2932 cm^−1^, 1644 cm^−1^, 1391 cm^−1^ 1097 cm^−1^, 617cm^−1^; and PL spectrum showed peak value at 445 nm (Figure 1c). The UV-vis absorption spectra of albumin–chlorogenic acid NPs were within the range of 200–1100 nm. Figure 1a shows that the UV-vis spectrum, in which broad absorption peaks at 257 and 292 nm were observed, confirmed the formation of albumin–chlorogenic acid NPs. FTIR spectroscopy is a suitable technique for determining the surface functionality of albumin–chlorogenic acid NPs, as shown in Figure 1b. The FTIR spectra for albumin–chlorogenic acid NPs showed multiple distinct bands at 3427, 2932, 1644, 1391, 1097, and 617 cm^−1^, respectively. The hydroxyl stretching O–H peak is observed at 3435 cm^−1^. The C–H asymmetric stretching at 2932 cm^−1^ and albumin molecular peaks at 1644 cm^−1^ were attributed to amide I (NH2) flexural vibration adsorption. The O–H bending of the phenol group was observed at 1391 cm^−1^ for the chlorogenic acid functional group. The C–O stretching length is 1097 cm^−1^. The overtone peaks are observed at 617 cm^−1^. This results in albumin conjugation with chlorogenic acid. The photoluminescence (PL) emission spectra of albumin–chlorogenic acid with an excitation wavelength are shown in Figure 1c. The deconvolution of the PL spectra of BNTC HNMs revealed seven peaks, which have been categorized as the PL spectrum at 366 nm; violet at 417 nm; blue at 439, 445, 455, and 479 nm; and green at 503 and 519 nm, respectively.

**Figure 2:**
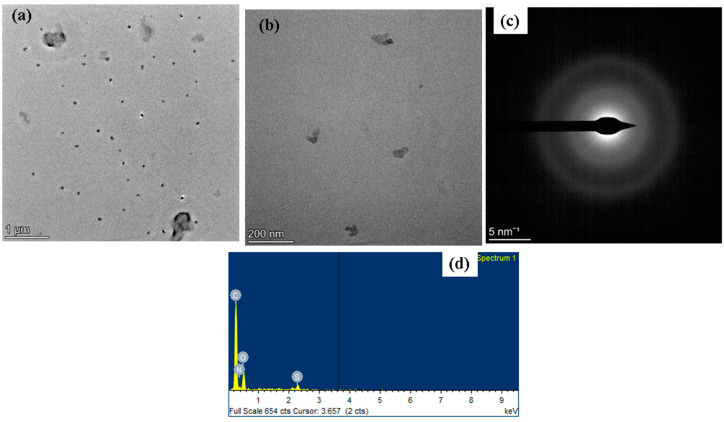
TEM micrographics of the albumin–chlorogenic acid NPs: lower and higher magnification TEM image (**a**–**c**). Elemental profile by EDX study (**d**).

The surface morphologies of albumin–chlorogenic acid NPs were examined using TEM and SAED at various magnifications (1 micrometer to 5 nanometers), as shown in Figure 2a–c. The TEM images show that the synthesized albumin–chlorogenic acid NPs have a spherical appearance. The average size is 20 nm. The surfaces of albumin are conjugated with chlorogenic acid. From the SAED pattern, the albumin–chlorogenic acid NPs made from albumin and chlorogenic acid display a SAED pattern and polycrystalline rings. An EDAX spectrum, as depicted in Figure 2d, revealed the chemical composition of synthesized albumin–chlorogenic acid NPs. The atomic percentages of the albumin–chlorogenic acid NPs were found to be C, N, O, and S, respectively.

**Figure 3:**
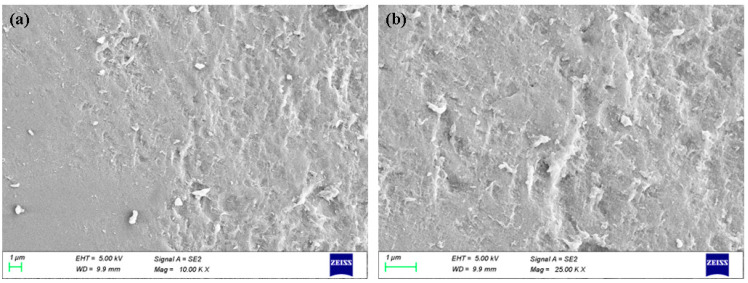
FESEM image of albumin–chlorogenic acid NPs. Lower (**a**) and higher (**b**) magnification.

Lower and higher magnification FESEM images were used to examine the surface morphologies of albumin–chlorogenic acid NPs, as shown in Figure 3a–b. The FESEM images show that the synthesized albumin–chlorogenic acid NPs have a spherical appearance. The average size is 20–30 nm. The surfaces of albumin are conjugated with chlorogenic acid.

**Figure 4:**
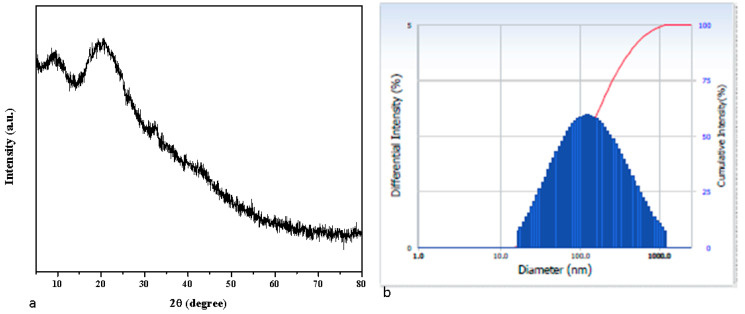
XRD Pattern (**a**) and DLS pattern (**b**) of albumin–chlorogenic acid NPs. Red line mention that cumulative intensity of the nanoparticles size.

The X-ray diffraction patterns of albumin–chlorogenic acid NPs. The peaks in the XRD spectrum of albumin–chlorogenic acid NPs witnessed their amorphous nature (Figure 4a). This decrease in crystallinity denotes that the amorphous properties of chlorogenic acid conjugated with albumin nanoparticles are present. The average crystalite size for albumin–chlorogenic acid NPs is 22 nm. As shown in Figure 4b, the hydrodynamic diameter of albumin–chlorogenic acid NPs was calculated using dynamic light scattering (DLS), and it was discovered to be 130 nm.

### 3.2. Analysis of Anti-Bacterial and Anti-Fungal Effects of Albumin–Chlorogenic Acid Green NPs

**Figure 5:**
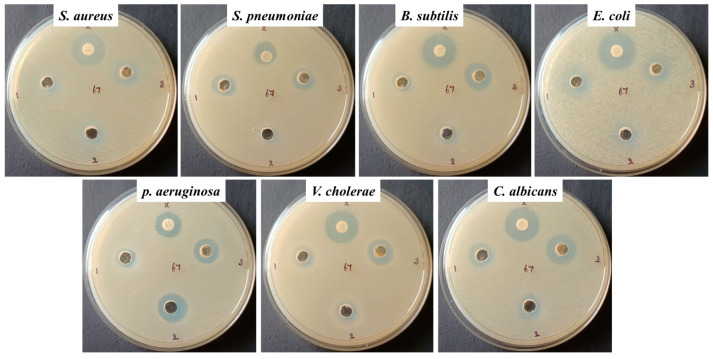
Antibacterial activity of albumin–chlorogenic acid NPs. NPs inhibit the growth of bacteria and fungi.

To determine the antimicrobial effects, albumin–CGA NPs at 1, 1.5, and 2 mg/mL and standard amoxicillin were used. *S. aureus* and *C. albicans*, and strains *S. pneumonia*, *B. subtilis*, *K. pneumonia*, *E. coli*, and *C. albicans* showed significant (*p* < 0.05) growth inhibition at 1.5 mg/mL in comparison with standard antibiotic amoxicillin. Where the growth inhibition at 2 mg/mL was almost similar to the standard drug amoxicillin and showed significantly (*p* < 0.05) less growth inhibition when compared to 1 and 1.5 mg/mL doses (Figure 5, Table 1).

### 3.3. Invitro Anti-Cancer Studies

#### 3.3.1. Cytotoxicity Assay

**Figure 6:**
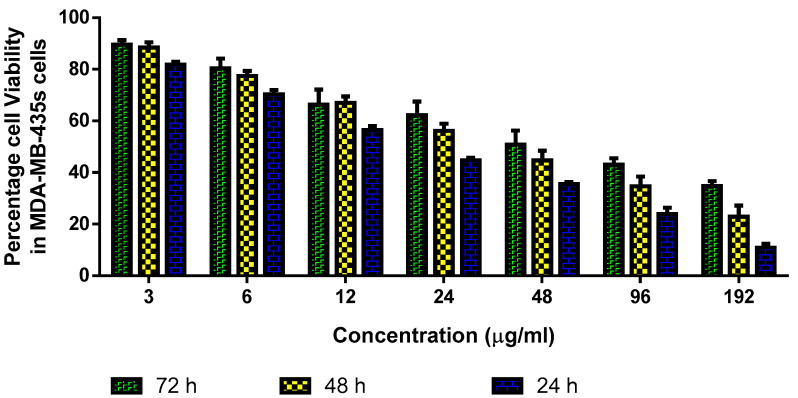
Albumin–chlorogenic acid NPs decreased the growth of MDA-MB-435s cells. Cells were treated with the diverse dosages (3–192 μg/mL) of albumin–chlorogenic acid NPs for 24, 48 and 72 h. The cell growth was examined by MTT assay and the outcomes are revealed as mean ± SD of triplicates.

MTT assay was done to investigate the cytotoxicity and inhibitory concentration 50 (IC50) value of albumin–CGA NPs (Figure 6). Treatment with albumin–CGA NPs for 24 h showed IC50 value at 26.65 μg/mL, whereas at 48 and 72 h of exposure. The MDA-MB-435s cells showed IC50 values at 15.81 and 8.83 μg/mL, respectively. For further subsequent experiments, dosage at 8.83 μg/mL was used as IC50 concentration, and the surviving cells were harvested and used for analysis of cell death and cell signaling cascade.

#### 3.3.2. Dual Staining

**Figure 7:**
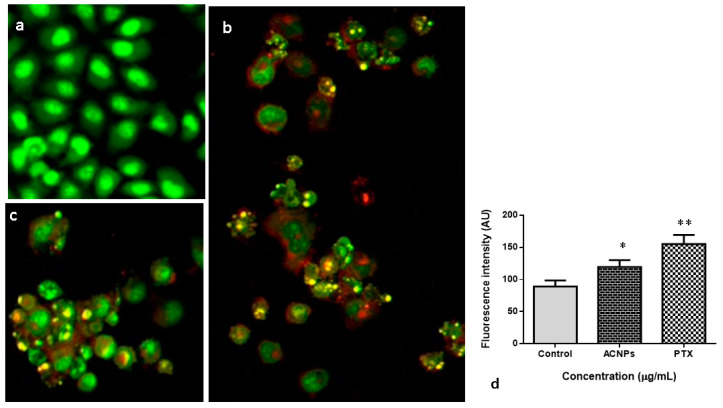
Effect of albumin–chlorogenic acid NPs on the apoptotic level in the MDA-MB-435s cells. Cells were stained using AO and EtBr (1:1) then investigated using fluorescent microscope. The green fluorescence was observed in control cells, which denotes the viable cells without apoptosis. The albumin–chlorogenic acid NPs (ACNPs) exposed cells revealed increased yellow/orange fluorescence that proves the onset of early and late apoptosis, respectively. (**a**) Control, (**b**) albumin–chlorogenic acid NPs-exposed cells ((ACNPs), IC50 concentration), (**c**) paclitaxel (0.5 μM) concentration, and (**d**) arbitrary units of fluorescent intensity was determined using fluorescent microplate reader. * *p* < 0.05 & ** *p* < 0.005 compared with control.

AO/EtBr analysis was performed to identify the number of MDA-MB-435s cells undergoing apoptotic upon treatment with albumin–CGA NPs (ACNPs; IC50 concentration) and paclitaxel (0.5 μM) for 24 h (Figure 7). Treatment with albumin–CGA NPs revealed a significant (*p* < 0.05) increase in AO/EtBr stained cells at IC50 concentration (albumin–CGA NPs). The results were similar to paclitaxel- (0.5 μM concentration) treated cells which showed significantly (*p* < 0.05) increased AO/EtBr stained cells. A significant level of AO/EtBr staining was not detected in the untreated cells.

#### 3.3.3. Cell Cycle Analysis

**Figure 8:**
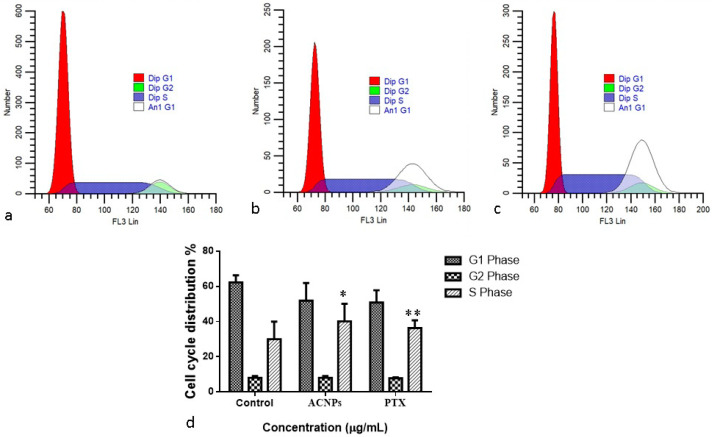
Cell cycle analysis using flow cytometry. MDA-MB-435s cells were treated with the IC50 dosage of albumin–chlorogenic acid NPs (ACNPs) for 24 h and paclitaxel (0.5 μM). Cell cycle pattern and apoptosis distribution; (**a**) Control, (**b**) albumin–chlorogenic acid NPs-exposed cells (ACNPs, IC50 concentration), (**c**) paclitaxel (PTX, 0.5 μM concentration), and (**d**) percentage of cell cycle distribution. Data are given as mean ± SD of triplicates. (* *p* < 0.05 & ** *p* < 0.001 when compared to control).

Figure 8 shows the findings of cell cycle analysis. MDA-MB-435s cells alterations in their cell cycle status upon treatment with albumin–CGA NPs (ACNPs; IC50 concentration) and paclitaxel (0.5 μM) for 24 h were evaluated by PI staining in the FACS machine. Treatment with albumin–CGA NPs showed changes in levels of G1 phase cells, whereas levels of cells in the S phase was substantially increased in the albumin–CGA NPs exposed group and paclitaxel (PTX)-treated group when compared to the total number of cells in control. This reveals replication arrest or inhibition in the S phase. While the total cell numbers were remarkably (*p* < 0.05 and *p* < 0.001) elevated in the S phase compared with G2/M phase in both PTX and albumin–CGA treated groups, respectively..

#### 3.3.4. Comet Assay Analysis

**Figure 9:**
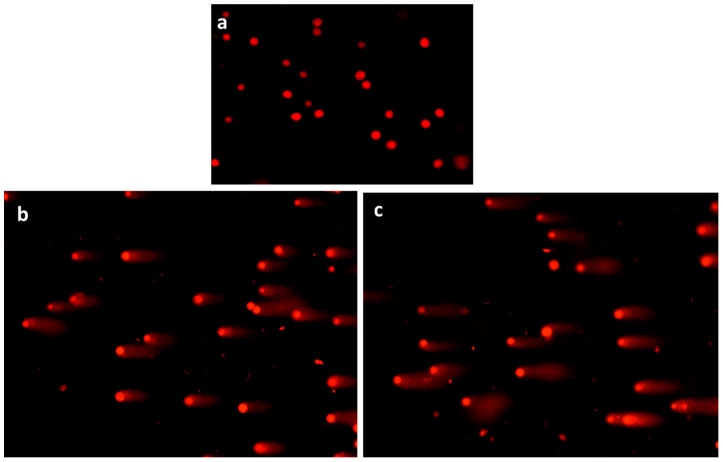
The DNA damage was measured by comet assay after treatment of MDA-MB-435s cells with albumin–chlorogenic acid NPs. The cells were (**a**) control (untreated cells), (**b**) albumin–chlorogenic acid NPs-treated cells (ACNPs, IC50 concentration), (**c**) paclitaxel (PTX, 0.5 μM concentration).

Figure 9 shows the status of comet assay analysis in MDA-MB-435s cells. Cells were exposed to albumin–CGA NPs (ACNPs; IC50 concentration) and paclitaxel (0.5 μM) treated cells were subjected to comet assay analysis. Treatment with NPs showed a significant increase in comet tail patterns in both albumin–CGA NPs (ACNPs; IC50 concentration) and paclitaxel- (0.5 μM) treated cells when compared to control or untreated cells.

#### 3.3.5. Annexin-V/FITC/PI Flow Cytometry

**Figure 10:**
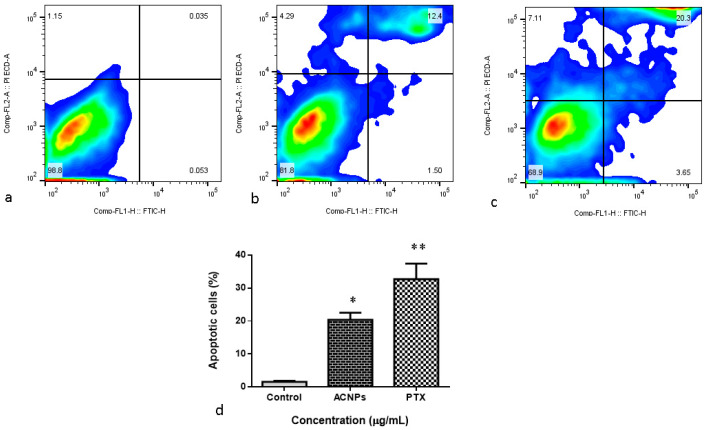
Annexin-V/-FITC/PI flow cytometry analysis of MDA-MB-435s cells treated with IC50 dosage of albumin–chlorogenic acid NPs for 48 h. (**a**) Control, (**b**) albumin–chlorogenic acid NPs-treated cells (ACNPs, IC50 concentration), (**c**) paclitaxel (PTX, 0.5 μM concentration), and (**d**) Early/late apoptotic cell percentage. The live, early apoptotic, and secondary necrotic cells were revealed by the lower left quadrant (Annexin-V À/PI À), lower right (Annexin-V +/PI À), and upper (Annexin-V+/PI+) quadrant, respectively. Data are given as mean ± SD of triplicates. (* *p* < 0.05 & ** *p* < 0.001 when compared to control).

Figure 10 illustrates the status of apoptosis in MDA-MB-435s cells upon albumin–CGA NPs (ACNPs; IC50 concentration) and paclitaxel (0.5 μM) administered to cells for 48 h. Albumin–CGA treated cells showed substantial upsurge in early apoptotic cells at 24 h, whereas at 48 h of treatment, the total number of early and late apoptotic cells were increased with the moderate number of necrotic cells. The positive control paclitaxel (0.5 μM) also showed similar results with ACNPs-treated cells compared with the control.

#### 3.3.6. Intracellular ROS Analysis by DCFH-DA

**Figure 11:**
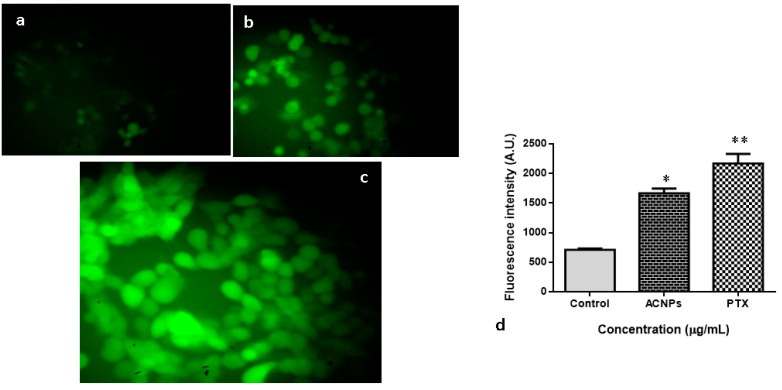
Endogenous ROS production induced by albumin–chlorogenic acid NPs stained with DCFH-DA. (**a**) Control, (**b**) albumin–chlorogenic acid NPs-exposed cells (ACNPs, IC50 concentration), (**c**) paclitaxel (0.5 μM) concentration, and (**d**) arbitrary units of fluorescent intensity were assessed by fluorescent microplate reader. * *p* < 0.05 & ** *p* < 0.001 compared with control. Magnification: 20×.

Figure 11 illustrates the status of intracellular ROS formation in MDA-MB-435s cells upon treatment with albumin–CGA NPs (ACNPs; IC50 concentration) and paclitaxel (0.5 μM). The MDA-MB-435s exposed to albumin for 24 h revealed a moderate increase in ROS formation. However, there is a considerable upsurge in a level of ROS at the ACNPs-treated group and PTX. ACNPs showed significant (*p* < 0.05) increase in ROS formation.

#### 3.3.7. Assay of Antioxidant Systems in MDA-MB435s Cells

**Figure 12:**
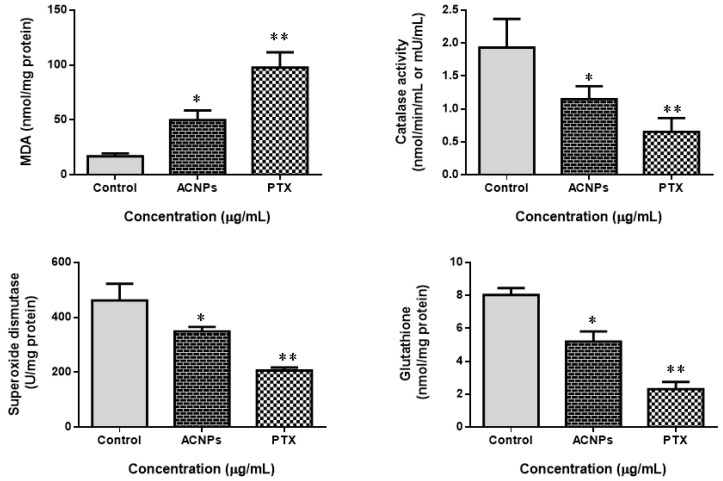
Effects of albumin–chlorogenic acid NPs on CAT, SOD, GSH, and MDA in MDA-MB-435s cells. Control, albumin–chlorogenic acid NPs-treated cells (ACNPs, IC50 concentration), paclitaxel (PTX, 0.5 μM concentration). CAT, SOD, GSH, and MDA: albumin–chlorogenic acid NPs. One unit of CAT activity is given as a quantity of protein that decomposes at 1 μmol H_2_O_2_/s. One unit for SOD activity is given as a quantity of protein needed for 50% inhibition of SOD activity where superoxide radicals oxidize hydroxylamine to produce nitrite. Data are exhibited as mean ± SD. * p < 0.05 & ** p < 0.001 compared with the control.

Figure 12 shows the status of cellular antioxidant in MDA-MB 435s cells treated with albumin–CGA NPs (ACNPs; IC50 concentration) and paclitaxel (0.5 μM). The MDA status was remarkably increased by ACNPs and PTX. Levels of SOD and CAT were substantially depleted in ACNPs and paclitaxel-exposed MDA-MB-435s cells. Similarly, the level of total cellular antioxidant GSH was also significantly (*p* < 0.05) reduced in ACNPs- and PTX-treated MDA-MB-435s cells compared to control/untreated cells.

#### 3.3.8. ELISA Analysis of Apoptotic Markers

**Figure 13:**
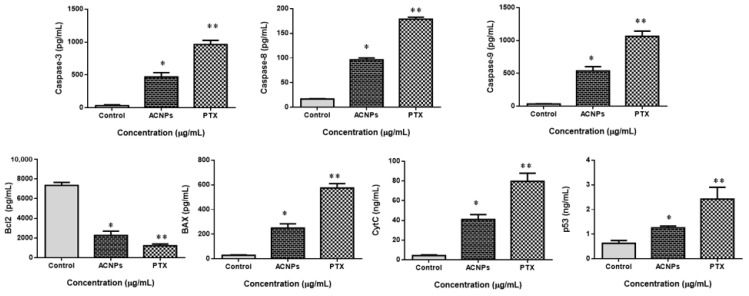
Albumin–chlorogenic acid NPs inhibits MDA-MB-435s cells (b) cell proliferation and promotes apoptosis. Levels of caspase-3, 8, 9, Bax, Bcl-2, CytC, and p53 in both cells were measured via ELISA. Control, albumin–chlorogenic acid NPs-exposed cells (ACNPs, IC50 concentration), paclitaxel (PTX, 0.5 μM concentration). Three independent repeated tests were conducted. The findings are portrayed as mean ± SD of triplicates. * p < 0.05 & ** p < 0.001 from the control.

Figure 13 shows the status of apoptotic protein levels in MDA-MB435s cells treated with albumin–CGA NPs (ACNPs; IC50 concentration) and paclitaxel (0.5 μM) for 24 h. Albumin–CGA treated cells exhibited a considerable upsurge in the caspase 3, 8, 9, Bax, CytC, and P53 protein levels, compared to control cells. Whereas the BCl-2 was significantly (*p* < 0.05) reduced in ACNPs and paclitaxel-treated MDA-MB435s cells when compared to control cells. The alteration in apoptotic proteins was significantly higher in ACNPs compared to the control group.

#### 3.3.9. Gene Expression Analysis of PI3K/AKT/mTOR Pathway

**Figure 14:**
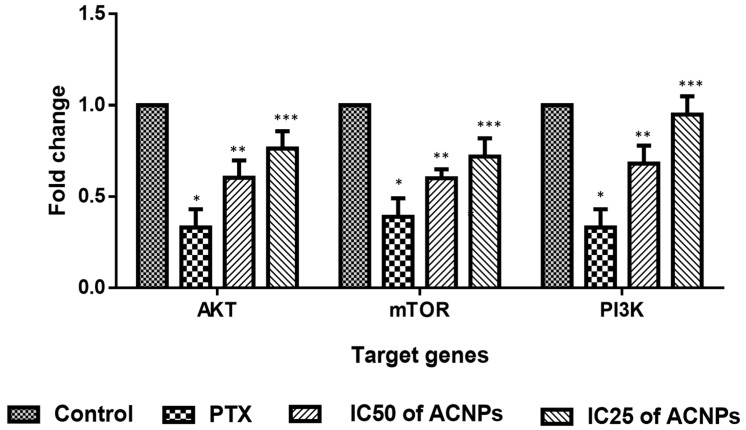
Effect of albumin–chlorogenic acid NPs on the PI3K/AKT/mTOR signaling in MDA-MB-435s cells. Control, albumin–chlorogenic acid NPs-treated cells (ACNPs, low dose: IC25 and high dose: IC50 concentration), paclitaxel (PTX, 0.5 μM concentration). Data are portrayed as mean ± SD of triplicates. * indicate the significance *p* < 0.01 compared with the control, ** indicate the significance *p* < 0.05 compared with the control and *** indicate the significance *p* < 0.001 compared with control.

Figure 14 shows the levels of PI3/AKT/mTOR protein levels in MDA-MB-435s cells administered with albumin–CGA NPs at (IC25 and IC50) and paclitaxel for 24 h. Albumin–CGA NPs treated cells showed significant AKT, mTOR, and PI3K protein levels when compared to control cells. The reduction in AKT, mTOR, and PI3K was substantially higher in IC50 than in the IC25 concentration-treated group.

## 4. Discussion

Our results showed that the synthesized albumin–CGA NPs exhibited typical biogenic green NP characteristics. Patterns observed from UV-vis spectrophotometer analysis, XRD analysis, PL spectrum, TEM and FESEM analysis, XRD, and DLS patterns all demonstrated significant results corresponding to the physiochemical nature of green biogenic nanoparticles reported previously using human serum albumin [34]. Next, we tested the anti-microbial effects of our biogenic green NPs albumin–CGA against both bacterial and fungal strains. Our results demonstrated that albumin–CGA exhibited significant cytotoxicity to various strains of bacteria (gram +ve and gram –ve) and fungal strains, ranging from 1 mg and 1.5 mg/mL concentrations. Notably, anti-microbial activity was observed up to 2 mg/mL concentration. This denotes that our albumin–CGA NPs probably penetrated the bacterial and fungus cell membranes via their affinity and small particle size. This would result in the intracellular generation of disproportionate ROS that could harm bacterial, and fungal metabolisms. However, the actual mechanism of bacterial uptake of nanoparticles is very limited due to the complex nature of rigid multilayer cell walls [35]. Here we speculate that our anti-microbial results are due to albumin–CGA NPs’ ROS generation and ROS-mediated damage to cellular molecules. This study’s results are similar to reports denoting the antimicrobial effects of albumin-assisted green NPs and CGA in various studies [36,37].

In this study, we used albumin to synthesize chlorogenic acid NPs. We have also characterized and tested its anti-microbial and anti-cancer activities in experimental in vitro models. Albumin is a globular protein and as a carrier protein (carrier of lipophilic substances like thyroid hormone, sex hormone, and triglycerides), it is used in the treatment of conditions like rheumatoid arthritis, diabetes, hepatitis, cancers, etc., [15]. Albumin is a versatile component in green NP synthesis. Due to its intrinsic nature as a component of blood, it is employed as a suitable agent for drug delivery systems in various disease conditions and also to overcome drug resistance mechanisms [16,17,18]. The presence of functional groups like the carboxyl group, an amino group, helps target ligands, as well as the fact that albumin’s stability favors enhanced drug delivery without degradation [12,13]. Hence, we have used albumin to conjugate chlorogenic acid (CGA) and to synthesize green albumin CGA NPs for evaluating its use in anti-microbial and anti-cancer applications. Chlorogenic acid (CGA) is a phenol component present in dietary components like cherries, citrus fruits, apples, carrots, tea, coffee, etc. [19]. CGA was reported to exert anti-oxidant, inflammatory-relieving, nephroprotective, hepatoprotective, and antibacterial functions [20,21].

Next, to determine the in vitro anticancer effects of our newly synthesized green NPs, albumin–CGA, we used MDA-MB435s cells. Cytotoxicity assay was performed using various doses for 24, 48, and 72 h in 96-well plates. Our results showed albumin–CGA NPs showed IC50 cytotoxicity at a concentration of 24 μg/mL for 24 h of treatment. Following this, for analysis of various oxidative stress parameters, cellular apoptosis mediator analysis, genome damage analysis, and cell signaling molecule analysis we have employed the IC50 and IC25 dosage to determine the dose-dependent cytotoxic effects of albumin–CGA for 24 and 48 h treatments based on the experimental studies. Analysis of apoptotic cell status in albumin–CGA treated groups using AO/EtBr analyses demonstrated considerable induction in AO and EtBr staining to cells at IC25 and IC50 which is similar to the standard drug paclitaxel (0.5 μM). This denotes our green NPs have a significant ability to damage the cell membrane, and thereby induce apoptotic/necrotic events. This could be attributed to its direct action on cellular macromolecules or via indirect effects via ROS-mediated oxidative damage to cellular metabolism [38]. Furthermore, cell cycle analysis revealed that albumin–CGA NPs treatment showed a significant reduction in S phase cells and significantly increased cell accumulation in the G2/M phase than G0/G1 phase. This denotes the inhibition of cell replication and activation of genome damage checkpoints. This could be due to albumin–CGA-mediated growth inhibition and genomic damage to the MDA-MB-435s cells. The results of our study were, in turn, similar to the previous report denoting the accumulation of more hepatocellular carcinoma cells in the G0/G1 phase [39]. Next, to confirm the induction of genomic damage as evident from the gathering of more cells in the G2/M checkpoint, we analyzed MDA-MB-435s cells via single-cell comet electrophoresis. Comet assay results revealed the induction of significant genomic damage at IC50 concentration in a dose-dependent manner. Similarly, our FACS analysis also revealed induction of apoptosis as observed from the occurrence of early and late apoptosis at IC50 concentration in a dose-dependent manner. These results together confirm the apoptosis-inducing ability of albumin–CGA NPs in our study. This is, in turn, supported by reports denoting that albumin NPs induce apoptosis in tumor cells [40].

To confirm whether the above apoptosis-inducing effects, cell cycle disruption, compromised membrane integrity, and altered cellular architecture were mediated by oxidative stress in the above experiments, we analyzed intracellular ROS in MDA-MB-435s cells. Our results demonstrated that albumin–CGA-treated cells produced disproportionate ROS at IC50 concentrations. Similarly, we have also analyzed the status of intracellular oxidants, GSH, and levels of antioxidant enzymes SOD, and CAT in MDA-MB-435s cells. Results showed albumin–CGA treated cells showed a significant reduction in GSH, SOD, and CAT status, and an upsurge in the MDA level. Altogether our results showed albumin–CGA NPs induce oxidative stress and compromise antioxidant defense. This could be because of the overproduction of ROS and utilization of cellular machinery to quench the same leading to deregulated metabolism and cell damage. Outcomes of our work highlight that the synergistic effect of albumin–CGA could cause enhanced ROS production resulting in oxidative stress [41,42]. In addition to the above, levels of apoptotic proteins caspase 3, 8, 9, proapoptotic Bax, Cyt C, and P53 were significantly increased, and anti-apoptotic Bcl-2 was significantly reduced in MDA-MB-435s cells treated with albumin–CGA NPs in a dose-dependent manner (IC25 and IC50). Along with this, we have also observed that PI3K/AKT/mTOR pathway activation was also significantly inhibited in MDA-MB-435s cells at IC25 and IC50 concentrations [43]. Altogether, our results denote, albumin–CGA NPs via their ability to induce ROS and penetrate tumor cell membranes induce oxidative stress, compromise antioxidant machinery, and activate intracellular apoptosis in a p53-dependent manner and thereby induce cytotoxicity to MDA-MB-435s cells. However, our results are preliminary and confined to in vitro conditions. Additionally, in vivo works are warranted to confirm albumin–CGA NP biological functions.

## 5. Conclusions

We have synthesized and characterized ACNPs using a variety of techniques to confirm their formation and investigate their antifungal, antibacterial, and anticancer properties. A sphere-shaped structure is observed in ACNPs, and their average crystallite size is 20 nm. Several bacterial strains such as *S. aureus*, *S. pneumonia*, *B. subtilis*, *E. coli*, *P. aeruginosa*, *V. cholera*, and *C. albicans* were susceptible to the antibacterial effects of ACNPs. Nanomaterials showed considerable cytotoxicity against MDA-MB-435s as a ductal carcinoma in breast cancer cells using the MTT test, the dual AO/EtBr assay, DCFH-DA, annexin-V/-FITC/PI, and PI staining methods. ROS levels in the cells increased as a result of ACNPs. PI3K/Akt/mTOR signaling cascade activation, membrane disruption, and antioxidant enzyme reduction led to cell death. Future research will examine the molecular mechanism underlying breast cancer cell death in vivo using ACNPs as strong antibacterial and anticancer therapy alternatives.

## Figures and Tables

**Table 1:** Antibacterial activity was determined for albumin–chlorogenic acid NPs by measuring zone of inhibition (mm).

Bacterial and Fungal Species	Zone of Inhibition (cm)			
	1 mg/mL	1.5 mg/mL	2 mg/mL	Amoxicillin/Amphotericin-B
*S. aureus*	9.5	10	17	19.5
*S. pneumoniae*	11.5	7	11.5	13
*B. subtilis*	10.5	8	14.5	18.5
*K. pneumoniae*	12	9	13.5	18
*E. coli*	19	10.5	19	22
*P. aeruginosa*	10.5	15	13.5	14.5
*C. albicans*	12.5	12.5	16	18.5

## Data Availability

We will be able to provide you with data upon request.
